# One-pot pyro synthesis of a nanosized-LiMn_2_O_4_/C cathode with enhanced lithium storage properties†

**DOI:** 10.1039/c9ra04015c

**Published:** 2019-08-02

**Authors:** Jeonggeun Jo, Sukyeung Nam, Seungmi Han, Vinod Mathew, Muhammad Hilmy Alfaruqi, Duong Tung Pham, Seokhun Kim, Sohyun Park, Sunhyun Park, Jaekook Kim

**Affiliations:** Department of Materials Science and Engineering, Chonnam National University 300 Yongbong-dong, Bukgu Gwangju 500-757 Republic of Korea jaekook@chonnam.ac.kr +82-62-530-1699 +82-62-530-1703

## Abstract

A simple one-pot polyol-assisted pyro-technique has been adopted to synthesize highly crystalline, carbon-coated LiMn_2_O_4_ (LMO/C) nanoparticles for use as a cathode material in rechargeable Li-ion battery (LIB) applications. The phase purity, structure and stoichiometry of the prepared cathode was confirmed using X-ray techniques that included high-resolution powder X-ray diffraction and X-ray absorption fine structure spectroscopy. Electron microscopy studies established that the synthetic technique facilitated the production of nano-sized LMO particles with uniform carbon coating. The prepared LMO/C cathode demonstrates excellent electrochemical properties (cycling stabilities of 86% and 77.5% and high rate capabilities of 79% and 36% within the potential windows of 3.3–4.3 V and 2.5–4.3 V, respectively). The high electrochemical performance of the LMO/C cathode is attributed to the nano-size of the LiMn_2_O_4_ particles enabling high surface area and hence greater lithium insertion and also the uniform amorphous carbon coating facilitating effective reduction in manganese dissolution and volume expansion during the lithium de-intercalation/intercalation reactions. In addition, cyclic voltametry and impedance characterization confirm the reversible Li-intercalation and the role of the solid electrolyte interface layer (SEI) in the stable electrochemical reaction of the LMO/C electrode. Furthermore, this study shows the efficacy of a simple and low-cost pyro-synthetic method to realize high performance nano-sized particle electrodes with uniform carbon coating for useful energy storage applications.

## Abstract 2

A simple one-pot polyol-assisted pyro-synthesized LiMn_2_O_4_/C cathode demonstrated remarkable lithium cycling stabilities of 86% and 19% and high rate capabilities of 77.5% and 36% at 3.3–4.3 V and 2.5–4.3V, respectively.

## Graphical abstract

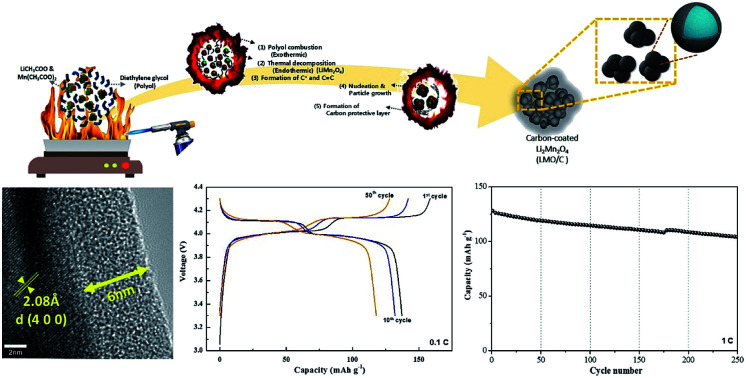

## Introduction

Nanomaterials have received much attention in various fields such as physical, chemical, biological, and material sciences, engineering, and electronics, and especially, for application in energy storage devices such as LIBs.^1–3^ The performance of a typical LIB is mainly determined by the cathodic material, which controls the energy density (capacity and voltage) and power density (rate capability).^4^ Therefore, the development of efficient cathodic materials is highly significant and challenging, and numerous studies are being carried out in this direction. Transition metal oxides have attracted much interest as cathode materials because they provide high potential and high reversible capacity, which are required for efficient lithium storage.^5^ Spinel LiMn_2_O_4_ is one of the most promising cathodic materials for Li-ion batteries because of its lower cost, environmental friendliness, and higher voltage than layered LiCoO_2_-like oxides.^6,7^ Importantly, the three-dimensional ion-diffusion in spinel LiMn_2_O_4_ facilitates a high theoretical capacity (∼148 mA h g^−1^).^8^ However, the manganese dissolution during electrochemical reaction and the kinetic issues related to the low electronic conductivity and lithium-ion diffusion coefficient and the rapid capacity fading during cycling of spinel LMO limit their practical application in high power LIBs. To suppress Mn dissolution many researchers are making great efforts to develop stable LMO materials by using various approaches, including electrolyte additives and surface coating strategy.^9–11^ Electrode performance, in general, is hugely influenced by the physico-chemical properties including compositional stoichiometry, particle size and distribution, particle orientation, crystallinity and morphology of the active material.^12,13^ Especially, particle size is critical to electrochemical performance because a high surface to volume ratio allows for a large electrode–electrolyte contact area.^14–20^ This implies that, despite the ion-migration not being rapid among the active particles, confining particle-size to the nano-scale regime is advantageous for performance due to the shortening of the ion-diffusion path. Also, utilizing nano-sized active materials with different dimensional (1D/2D/3D) structures is one of the effective ways to improve the performance of LMO for LIBs.^21–24^ In short, electrodes made from nanomaterials are expected to be widely used in next generation power sources. Further, the targeted stoichiometric, particle dimension, structural and morphological compositions of the LMO nanomaterials are determined by the material syntheses. Literature indicates that various synthetic techniques including high-energy milling,^24^ sol–gel processes,^12,25^ combustion,^26,27^ hydrothermal,^14^ precipitation,^28^ and ultrasonic spray pyrolysis^29^ were used to prepare nanosized LMO cathodes for LIBs. Each of these methods have their own advantages and disadvantages. However, most of these techniques involve either costly and complicated set-ups or complex and/or multi-step procedures,^30^ which are difficult to be employed for mass production and commercialization.^31^ Although combustion methods are attractive, from the economic standpoint, only a few of these techniques, particularly based on solution combustion and spray pyrolysis methods and the corresponding variants including flameless combustion and flame or ultrasonic spray pyrolysis were reported.^32–37^ Specifically, to produce LMO nanoparticles at apparently lower temperatures compared to few of the known syntheses, combustion techniques are considered more effective as LMO can be prepared at apparently lower temperatures (∼200–400 °C).^38^ Considering the effectiveness of these strategies related to LMO cathode synthesis and their persistent issues of electrochemical kinetics and capacity fade in LIBs, the exploitation of new designs or customizations to control the dynamic parameters of the combustion process can be highly significant for effective industrialization.

In the recent past, we introduced a polyol-assisted pyro-technique as a new combustion-based synthesis for the production of functional nanocrystals in very short reaction times of a few seconds in an open-air atmosphere at ambient temperatures.^39–45^ This polyol-assisted pyro-technique is based on a combination of different strategies, using a low-cost polyol solvent that acts as a capping agent, carbon source, and fuel for sustained combustion during nanoparticle synthesis. This technique exploits the ultrahigh energy released from the combustion of polyol for precursor decomposition, nucleation, and particle growth. Utilizing this technique, we successfully synthesized high performance LiFePO_4_/C, one of the recently commercialized cathode for LIBs. Although our earlier study was centered on introducing a new synthetic method to develop various nanomaterials, an in-depth investigation on the electrochemical properties specific to LMO/C cathodes prepared by this pyro-technique was not attempted.

In the light of the above discussions, we synthesized nanosized LMO/C *via* the polyol-assisted pyro-technique in the present study and investigated their cathode performance in LIB applications.

## Experimental section

### Synthesis of nano-LiMn_2_O_4_/C

**Scheme 1:**
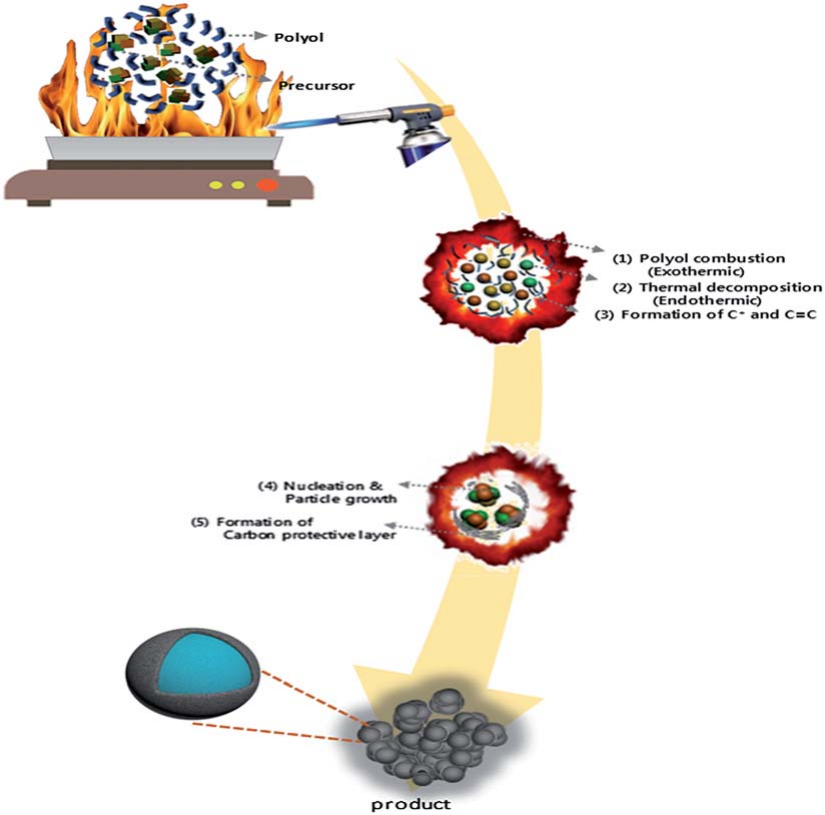
Schematic diagram of the polyol-assisted pyro synthesis to produce nano-sized LMO/C cathode.

LMO/C with the spinel structure was synthesized by a polyol-assisted pyro-synthesis method (Scheme 1) as follows. First, Li-acetate (Li-CH_3_COO) and Mn-acetate (Mn-(CH_3_COO)_2_) were dissolved in 80 mL of diethylene glycol (DEG) in the molar ratio 1 : 2 (Li : Mn). Subsequently, an inflammable liquid thinner (50 mL) was added to the precursor solution, and then stirred for 30 min. The final solution was uniformly poured onto a hot plate maintained at 350 °C. The solution was then ignited with an electric torch, which led to fast precipitation. The powders obtained after self-extinguishing were heat-treated at 800 °C for 10 h in open air to obtain highly crystalline samples.

### Structure and morphology characterization

A Shimadzu X-ray diffractometer with Ni-filtered Cu Kα radiation (*λ* = 1.5406 Å), operating at 40 kV and 30 mA, was used to record the powder X-ray diffraction (XRD) patterns in steps of 0.02° within the scanning angle range (2*θ*) of 10–80° for the prepared samples. The synchrotron XRD (SXRD) data were collected at the 9B high-resolution powder diffraction beamline of the Pohang Accelerator Laboratory (PAL), Korea. The SXRD pattern was fitted using the FULLPROF program for accurate structural determination.

The surface morphology of the samples was analyzed by field-emission scanning electron microscopy (FE-SEM, HITACHI S-4700) and field-emission transmission electron microscopy (FE-TEM, FEI Tecnai F20) at the Korea Basic Science Institute (KBSI). The carbon contents in the prepared samples were determined with an elemental analyzer (EA-1110, Thermo Quest, Italy). X-ray photoelectron spectroscopy (XPS) studies using a Thermo VG Scientific instrument (Multilab 2000) model was also performed to estimate the carbon content in the prepared sample. The elemental oxidation states and local structural properties were examined by X-ray absorption fine structure (XAFS) spectroscopy. The synchrotron X-ray absorption near edge structure (XANES) measurements were carried out at the BL7D beamline of the Pohang Light Source (PLS). All spectra were collected at room temperature in the transmission mode at the Mn K-edge. The collected data were processed using the normal method by obtaining the absorbance and analyzed using the ATHENA program.

### Electrochemical characterization

For the electrochemical measurements, the prepared LMO/C powder was mixed with 10 wt% of conducting carbon (Ketjen black EC300J, Lion Corporation, Tokyo, Japan) and 10 wt% of a polyvinylidene fluoride (PVDF) binder. The amount of conducting carbon was determined by taking into account the carbon content present in the active material; the active material loading was 3.5 mg cm^−2^. The mixture was then pressed onto a stainless steel mesh and vacuum-dried at 120 °C for 12 h to obtain the electrode. A 2032 coin-type cell with the LMO/C-based electrode and a Li metal anode separated by a polymer membrane was fabricated in an Ar-filled glove box and aged for 12 h. The electrolyte employed was a 1 : 1 mixture of ethylene carbonate (EC) and dimethyl carbonate (DMC) containing 1 M LiPF_6_ (PuriEL, Soul Brain, South Korea). Galvanostatic tests were carried out at room temperature using BTS 2004H (Nagano, Japan) at 3.3–4.3 V and 2.5–4.3 V, respectively.

## Results and discussion

**Fig. 1:**
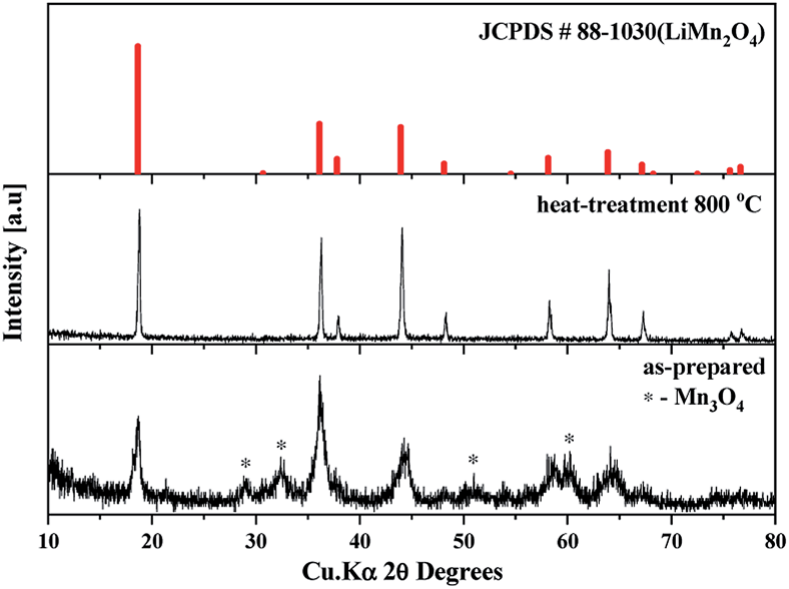
Schematic diagram of the polyol-assisted pyro synthesis to produce nano-sized LMO/C cathode.

**Fig. 2:**
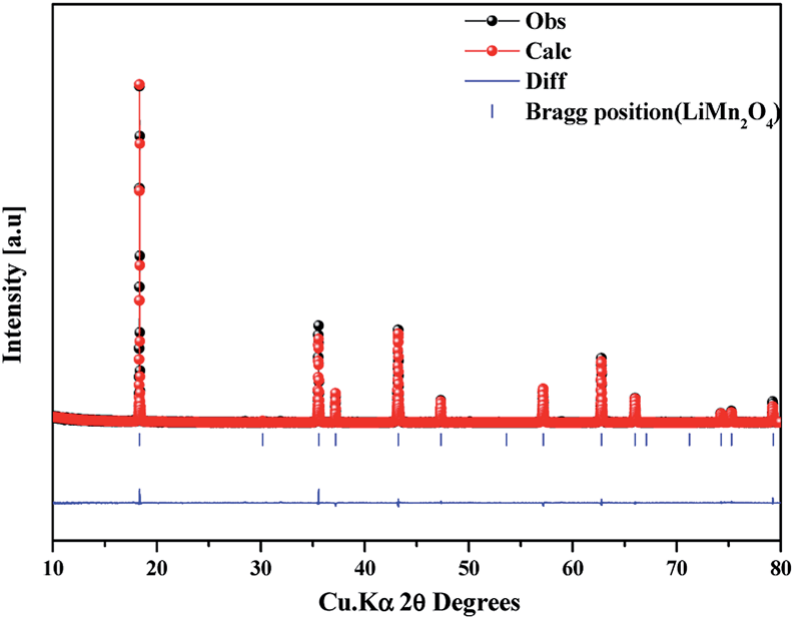
Synchrotron powder XRD profile and Rietveld refinement pattern of the LMO/C sample prepared by the polyol-assisted pyro.

The as-prepared sample obtained by polyol-assisted pyro-synthesis in a very short time was calcined at 800 °C in an open-air atmosphere for a short duration of 10 h to produce highly crystalline particles. The XRD pattern of the as-prepared sample is presented in Fig. 1. The sample shows nanoparticle characteristics, as indicated by the broad peaks. In addition, the sample shows composite formation, as indicated by the LiMn_2_O_4_ and Mn_3_O_4_ peaks. However, after heat treatment at 800 °C in an open-air atmosphere, as expected, the sample shows highly intense reflection lines corresponding to the pure, single phase, which is modeled as the spinel phase (space group: *Fd*3̄*m*). The absence of impurities in the recorded patterns indicates the formation of the pure crystalline phase. To gather detailed information of the structure, Rietveld refinement was performed on the SXRD pattern of the prepared LMO/C composite using the FULLPROF method, and the obtained pattern and refinement data are presented in Fig. 2 and Table 1, respectively. The results clearly confirm the cubic structure of the LMO phase. The lattice parameter value is determined to be 8.2369 Å (*a* = *b* = *c*). It is known that the manganese valence state of the high temperature spinel can slightly vary depending on the synthetic or calcination temperature.^46^ Precisely, the average manganese oxidation state of Mn^4+^ in LiMn_2_O_4_ decreases upon increasing calcination temperatures (500–900 °C). In other words, at high temperatures, the lowered average manganese oxidation states suggests that more amount of unstable Mn^3+^ ions exist than Mn^4+^ ions. This implies that LMO spinel unit cell formed at higher temperatures display a slightly increasing lattice parameter value. Given that the present spinel compound is prepared at 800 °C, the higher lattice parameter value (>∼8.17 Å) suggest the presence of higher amount of Mn^3+^ ions then the Mn^4+^ ions in the spinel structure. Furthermore, this indicates a low amount of cation vacancies and similarity in cation distribution between the tetrahedral and octahedral sites.^46^ More importantly, the “goodness of fit” (low *R*_p_, *R*_wp_, and *R*_exp_ values, as shown in Table 1) clearly validates the refinement process.

**Table 1:** Rietveld refinement data of the LMO/C sample prepared by the polyol-assisted pyro synthesis^a^

	Atom	Site	Wyckoff position			Occupancy
LiMn_2_O_4_	Li1	8a	0.125	0.125	0.125	0.25
	Mn	16d	0.5	0.5	0.5	0.5
	O	32e	0.26413	0.26413	0.26413	1

a *R*_p_ = 9.68, *R*_wp_ = 13.9, *R*_exp_ = 10.33, chi^2^ = 1.80, *S* = 1.4.

The average crystallite sizes were calculated from the XRD patterns using the first few highly intense diffraction planes of (1 1 1), (3 1 1), (4 0 0), according to the Scherrer formula (eqn. (1)). The calculations reveal that the average crystallite size range between 10 and 100 nm for both the as-prepared and heat-treated samples. It is important to note here that the exact estimation of the crystallite sizes requires the use of additional instrumental broadening data in eqn (1).1
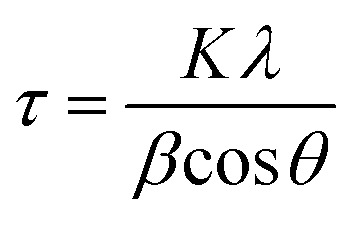
where *τ* = particle size, *K* = shape factor (0.98 for spherical particle), *λ* = X-ray wavelength (Cu Kα = 1.5406 Å), *β* = line broadening at half the maximum intensity (FWHM), and *θ* = Bragg angle.

**Fig. 3:**
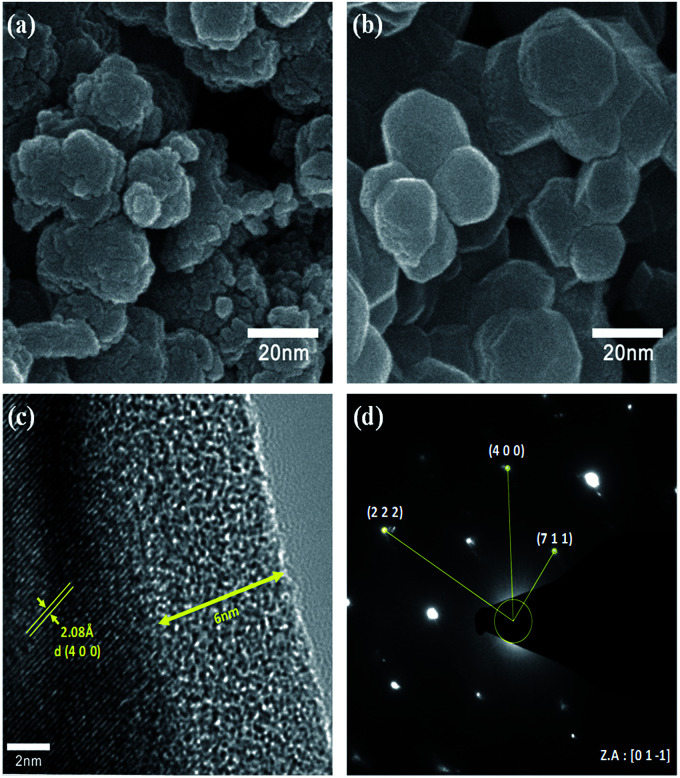
FE-SEM images of the LMO/C sample (a) as-synthesized, (b) calcined at 800 °C, (c) high-resolution-TEM image of the calcined LMO/C prepared by the polyol-assisted pyro synthesis and (d) SAED pattern index with LMO.

To verify the particle size calculated using the Scherrer formula, we conducted FE-SEM analysis. The FE-SEM images (Fig. 3) reveal the average primary particle sizes to be ∼10 nm and ∼30 nm for the as-prepared and heat-treated samples, respectively. Thus, the particle size calculated using the Scherrer formula is in accordance with the actual particle size. Also, the larger particle size for the heat-treated sample indicates particle growth during the heat treatment. Moreover, the overall morphology of both the samples show some amount of particle aggregation. Nonetheless, it is worth noting that the prepared samples can contain amorphous carbon due to the usage of polyol (fuel), which acts as the carbon source in the combustion process during the synthesis. C1s XPS spectra, which is provided in the ESI (Fig. S1†), confirmed the presence of carbon in the prepared LMO/C sample. As expected, the pattern revealed peak locations at 284.6 eV (C–C), 285.8 eV (C–O) and 288.6 eV (C=O) thereby confirming organic groups terminated on the surface and facilitating the subsequent carbon coating. From elemental analysis, the practical carbon contents in the as-prepared and calcined samples were estimated to be 5.4% and 0.44%, respectively. In addition, carbon can be present in the amorphous phase in the samples as the corresponding XRD patterns did not reveal carbon peaks. The carbon loss during heat treatment in air can be attributed to the formation of carbon dioxide (CO_2_). Further, the estimated amount of carbon is considered to be sufficient for carbon coating and/or network formation in the prepared samples. To confirm this phenomenon, we performed FE-TEM analysis on the heat-treated LMO/C sample. The FE-TEM image, which shows the carbon coating and the lattice fringes, and the corresponding SAED pattern are shown in Fig. 3. The TEM image in Fig. 3(c) shows the ∼6 nm-thick amorphous carbon coating on the particle surface. In addition, the lattice fringes can be well-indexed to the (4 0 0) lattice planes with a *d*-spacing of 2.08 Å. Moreover, the corresponding SAED pattern, which is shown in Fig. 3(d), reveals the (2 2 2), (4 0 0), and (7 1 1) Miller indices identified along the [0 1 −1] zone axis. These findings consider the facts that a fair distribution of carbon is observed and indicating the formation of highly crystalline LMO particles. The carbon coating/network acts as an electrical conduit between the particles, causing an improvement in the electrochemical properties at even high charge/discharge rates.^39^ The above results thus clearly suggest the successful production of highly crystalline LMO/C containing a carbon network/coating by an efficient one-pot process or pyro-synthetic strategy and an increase the overall electronic conductivity of the prepared material.

**Fig. 4:**
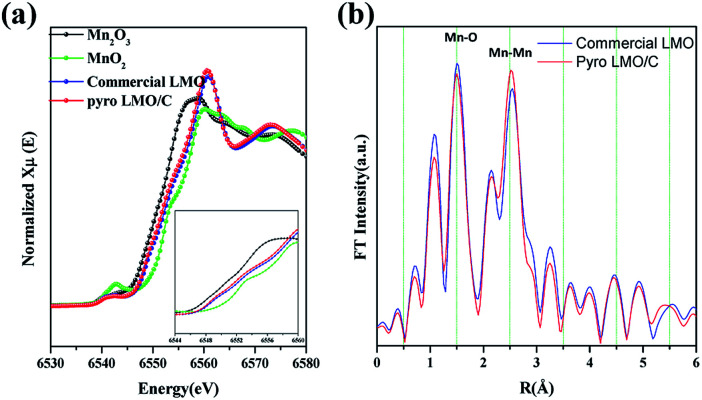
(a) Normalized Mn K-edge XANES spectra of the nanosized LMO/C compared to those of standard Mn_2_O_3_, MnO_2_ and commercial LMO powders. Inset shows close-up view of the white-line features of the spectra and (b) the corresponding Fourier Transforms (FTs) of the Mn K-edge EXAFS spectra in nanosized LMO/C and commercial LMO.

To confirm the oxidation state of Mn, we performed synchrotron XANES spectroscopy. Fig. 4 compares the spectrum of LMO/C with that of Mn_2_O_3_ (reference Mn(iii) state) and MnO_2_ (reference Mn(iv) state). Interestingly, the XANES patterns of the LMO and LMO/C samples are located between Mn_2_O_3_ and MnO_2_ reference spectra, indicating that the average oxidation state of Mn in the samples is mixed Mn(iii) and Mn(iv). The step edge value (*E*_0_) is determined to be 6559.4 eV for commercial LMO and 6559.0 eV for the calcined LMO/C sample. This value indicates that the amount of Mn^3+^ ions is higher than that of Mn^4+^ ions in the LMO/C sample, as revealed by the Rietveld refinement analysis.

EXAFS spectroscopy was performed to analyze the local structure of LMO/C. The first peak at around 1.5 Å in the Fourier-transform (FT) spectrum corresponds to the Mn–O interaction in the first coordination sphere, and the second peak at around 2.5 Å is due to the Mn–Mn interaction in the second coordination sphere. In addition, the peak at around 4.5 Å represents the second Mn neighbor at the central Mn atom, and the peak at around 5.5 Å is due to multi-scattering caused by the Mn atom at twice the distance of the nearest Mn atom.^47^ Considering the size effect, the effect of size reduction is clearly observed as a progressive decrease in the peak amplitude with increasing distance. This phenomenon is related to the number of coordinated atoms. The low average particle-size in the nano-sized LMO/C sample leads to the presence of a higher number of under-coordinated atoms on the crystallites surface than those in the bulk material. Hence, the average coordination number of the present LMO sample is reduced.^48^

**Fig. 5:**
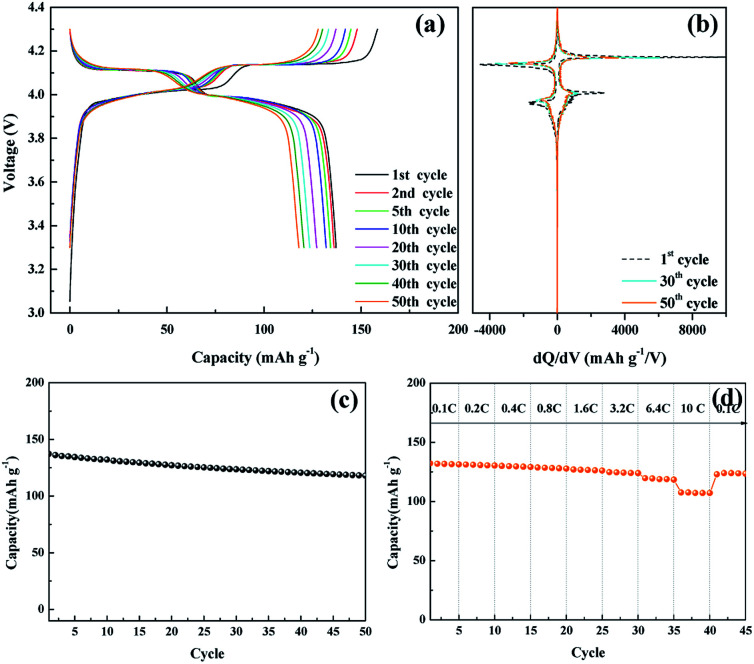
(a) Galvanostatic charge/discharge potential profiles of the nanosized LMO/C sample, (b) d*Q*/d*V* plot, (c) cycle performance, (d) C-rate performance at various current densities in the voltage range 3.0 to 4.3 V.

**Fig. 6:**
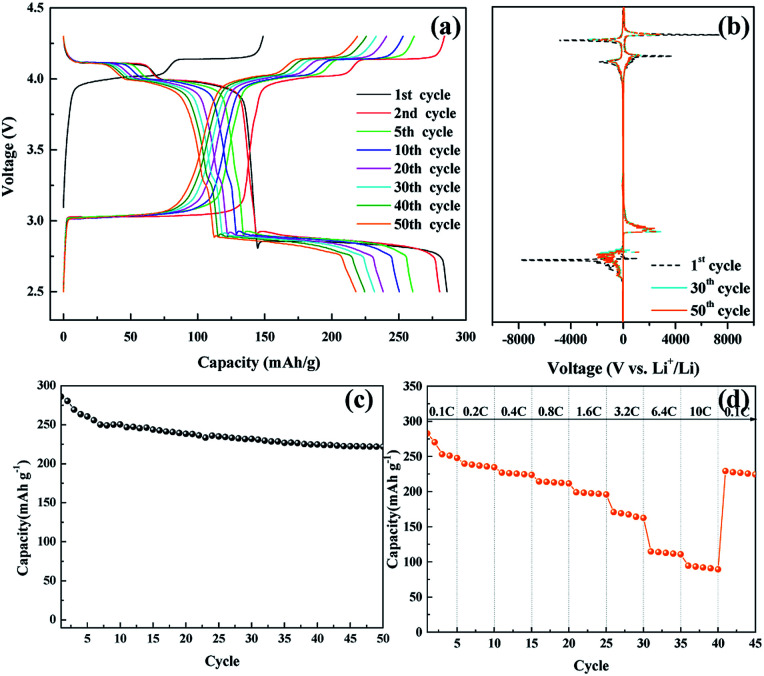
(a) Galvanostatic charge/discharge potential profiles of the nanosized LMO/C sample, (b) d*Q*/d*V* plot, (c) cycle performance, (d) C-rate performance at various current density in the voltage range 2.5 to 4.3 V.

The electrochemical performance of the nanosized LMO/C sample was measured in two different voltage ranges: 3.3–4.3 V (Fig. 5) and 2.5–4.3 V (Fig. 6). Fig. 5 shows the voltage profile for the nanosized LMO/C sample at a current density of 0.1C (0.1 mA) in the potential range of 3.3–4.3 V. The initial charge capacity is as high as 158.46 mA h g^−1^ and the discharge capacity is 137.2 mA h g^−1^. This voltage profile shows two pairs of redox plateaus during the charge and discharge states. The plateaus in the charge–discharge curves are transformed into peaks in the d*Q*/d*V* plots, as shown in Fig. 5(b). The LMO/C sample exhibits two well-defined splitting anodic/cathodic peaks, indicative of the spinel crystal structure. Two pairs of redox peaks are located at around 4.13/4.11 V and 4.02/3.98 V *vs.* Li^+^/Li, indicating that the reversible electrochemical de-intercalation/intercalation of Li^+^ from the tetrahedral sites of LMO occurs in two stages. The first redox peaks at around 4.0 V are ascribed to the insertion/removal of Li^+^ from half of the tetrahedral sites in which Li–Li interactions exist, whereas the second redox peaks at around 4.1 V are attributed to the insertion/removal of Li^+^ from the other tetrahedral sites without the Li^+^ ion interactions.^49^ Furthermore, the d*Q*/d*V* curves of different consecutive cycles overlap, indicating the consistent and stable electrochemical behavior of the spinel LMO/C sample. The cycle performance of the LMO/C electrode at 0.1C also shows the stable electrochemical behavior (Fig. 5(c)). The electrode exhibits a discharge capacity of 118.0 mA h g^−1^ after 50 cycles, which corresponds to 86% of its initial discharge capacity. Further cycle tests at a higher current density of 1C shows that 128 mA h g^−1^ initial capacity with 81% capacity retention after 250 cycles is achieved by the LMO/C cathode (Fig. S2†). A rate capability test was also performed on the electrode at different current rates between 0.1C and 10C by cycling it five times at each rate, as shown in Fig. 5(d). The electrode exhibits average discharge capacities of 135, 130, 126, and 119 mA h g^−1^ at current rates of 0.1, 0.4, 1.6, and 6.4C, respectively. Moreover, when cycled at a very high current rate of 10C, the electrode delivers a discharge capacity of 107 mA h g^−1^. It is also worth mentioning that the electrode discharge capacity recovers to 123 mA h g^−1^ (91% of its average initial discharge capacity) when the current rate reverts to 0.1C.

Fig. 6(a) shows the voltage profile of the nanosized LMO/C sample at the current density of 0.1C and potential range of 2.5–4.3 V, which corresponds to the de-intercalation/intercalation reaction of 2 Li^+^ ions. The initial charge capacity is as high as 148.8 mA h g^−1^ and the discharge capacity is 285.95 mA h g^−1^. The voltage profile shows three pairs of redox plateaus during the charge and discharge states, respectively. The plateaus in the charge–discharge curves are transformed into peaks in the d*Q*/d*V* plots, as shown in Fig. 6(b). The LMO/C sample exhibits three well-defined splitting anodic/cathodic peaks, indicative of the spinel crystal structure. In addition to the usual two redox peaks at high potentials, new redox current peaks are observed at 3.04/2.85 V, which correspond to the intercalation of Li^+^ ions at the octahedral 16c sites in cubic Li_*x*_Mn_2_O_4_ to form tetragonal Li_2_Mn_2_O_4_.^50^ Furthermore, the d*Q*/d*V* curve of different consecutive cycles overlap, and the peak intensity rapidly decays with increase in the cycle number. This result indicates that the unstable electrochemical behavior of the spinel LMO/C sample is due to Jahn–Teller distortion. The cycle performance of the LMO/C electrode at 0.1C is shown in Fig. 6(c). The electrode exhibits a discharge capacity of 221.8 mA h g^−1^ after 50 cycles, which corresponds to 77.5% of its initial discharge capacity. The rate capability test was also performed on the electrode at different current rates between 0.1C and 10C by cycling it five times at each rate, as shown in Fig. 6(d). The electrode exhibits average discharge capacities of 253, 225, 197, and 112 mA h g^−1^ at current rates of 0.1, 0.4, 1.6, and 6.4C, respectively. When cycled at a very high current rate of 10C, the electrode delivers a discharge capacity of 92 mA h g^−1^. It is also worth mentioning that the electrode discharge capacity recovers to 226 mA h g^−1^ (89% of its average initial discharge capacity) when the current rate reverts to 0.1C.

**Fig. 7:**
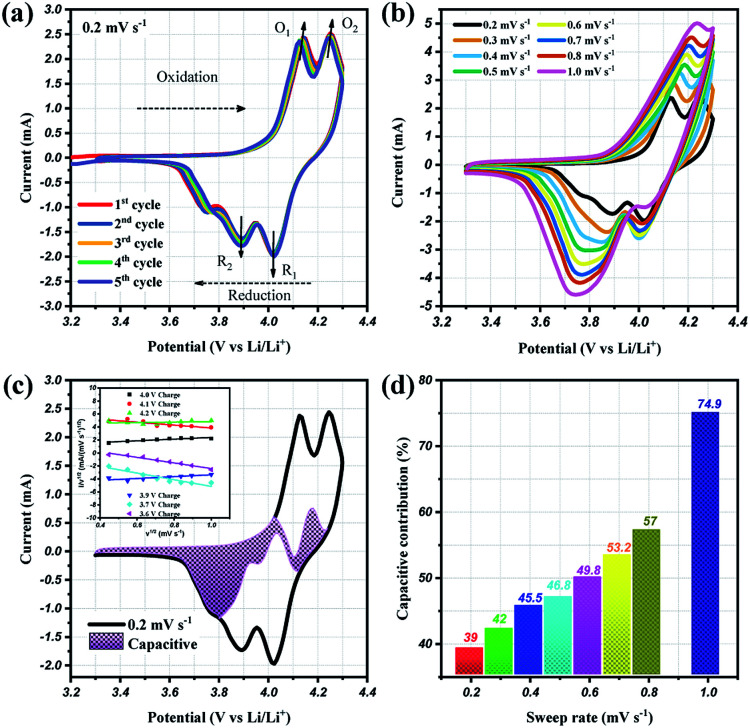
Cyclic voltammetry curves at (a) 0.2 mV s^−1^ and (b) different scan rates from 0.2 to 1.0 mV s^−1^, (c) CV curves showing the separation between total current (solid line) and capacitive contribution (shaded regions) at 0.2 mV s^−1^, inset: linear plots of cycle sweep-rate *vs.* current at different potentials to determine the slope constants in eqn (2) of text; (d) comparison of the capacitive storage distribution at different scan rates.

To prove the excellent cyclablilty and high rate performance, the electrochemical behavior of LMO/C as a cathode in LIB was further investigated by complex cyclic voltammetry analysis within the 3.3 V and 4.3 V potential domain. The CV profiles of the initial four cycles at 0.2 mV s^−1^, in Fig. 7(a), displays two pairs of redox peaks located around 4.25/4.02 V and 4.14/3.89 V *vs.* Li^+^/Li; the peak potentials are congruent with those in the d*Q*/d*V* result in Fig. 5(b), as expected. Further analysis on the cycle voltammetry curves were performed in order to understand the effect of capacitive behavior in determining the electrochemical performance. Fig. 7(b) illustrates the CV curves for the selected sweep rates from 0.2 to 1.0 mV s^−1^. As the scan rates were increased, the curve shape follows a similar trend, with steady peak shifts. The observed peak split mainly suggests a potential drop in the system due to the sluggish electron transport or electrode–electrolyte contact phenomenon.^51,52^ The area under the curve represents a combination of faradaic and double-layer non-faradaic charge storage mechanisms. Moreover, the faradaic mechanism represents two components: (i) Li^+^ ion insertion, and (ii) charge-transfer involving the surface particles or the so-called pseudocapacitance effect.^53–55^ The capacitive term is used to describe all the surface charge storage effects that include both the double layer and the pseudocapacitance charges. The power law relationship (*i* = *av*^*b*^) between the current (*i*) and the sweep rates (*v*) allows to qualitatively estimate the capacitive distribution.

Utilizing these concepts, Dunn and co-workers have developed an effective calculation method to provide further analysis *i.e.*, qualify capacitive and diffusion controlled contributions, and quantify their values related to the overall current.^56,57^ Based on the earlier discussion, the current response could be separated into surface-capacitive (*k*_1_*v*) and diffusion-controlled (*k*_2_*v*^1/2^) effects, represented by the following eqn (2):

2 *i* (V) = *k*_1_*v* + *k*_2_*v*^1/2^ or *i* (V)/*v*^1/2^ = *k*_1_*v*^1/2^ + *k*_2_

By plotting the square root of the sweep-rate dependence of current, *k*_1_ and *k*_2_ can be determined from the slope and *y*-axis intercept. The individual cathodic/anodic scan potential can be used to determine the *k*_1_ and *k*_2_ values. The *k*_1_*v*^1/2^ term enables us to clarify the capacitive current contribution along with the working potential range. The inset of Fig. 7(c) presents examples of the linear relationship between *v*^1/2^ (*x*-axis) and (*i*/*v*)^1/2^ (*y*-axis). At a given selected potential, *k*_1_ and *k*_2_ could be determined from the slope and intercept point of the linear line, respectively. Following the calculation, the capacitive distribution is estimated for the whole potential range and plotted in Fig. 7(c) for 0.2 mV s^−1^ sweep rate (shaded region), as an example. The comparison of the shaded area with the overall storage charge estimates that capacitive distributions are nearly 39%, 42%, 45%, 46%, 53%, 57% and 75%, at 0.2, 0.3, 0.4, 0.5, 0.6, 0.7, 0.8 and 1.0 mV s^−1^ scan rates, respectively (Fig. 7(d)).

**Fig. 8:**
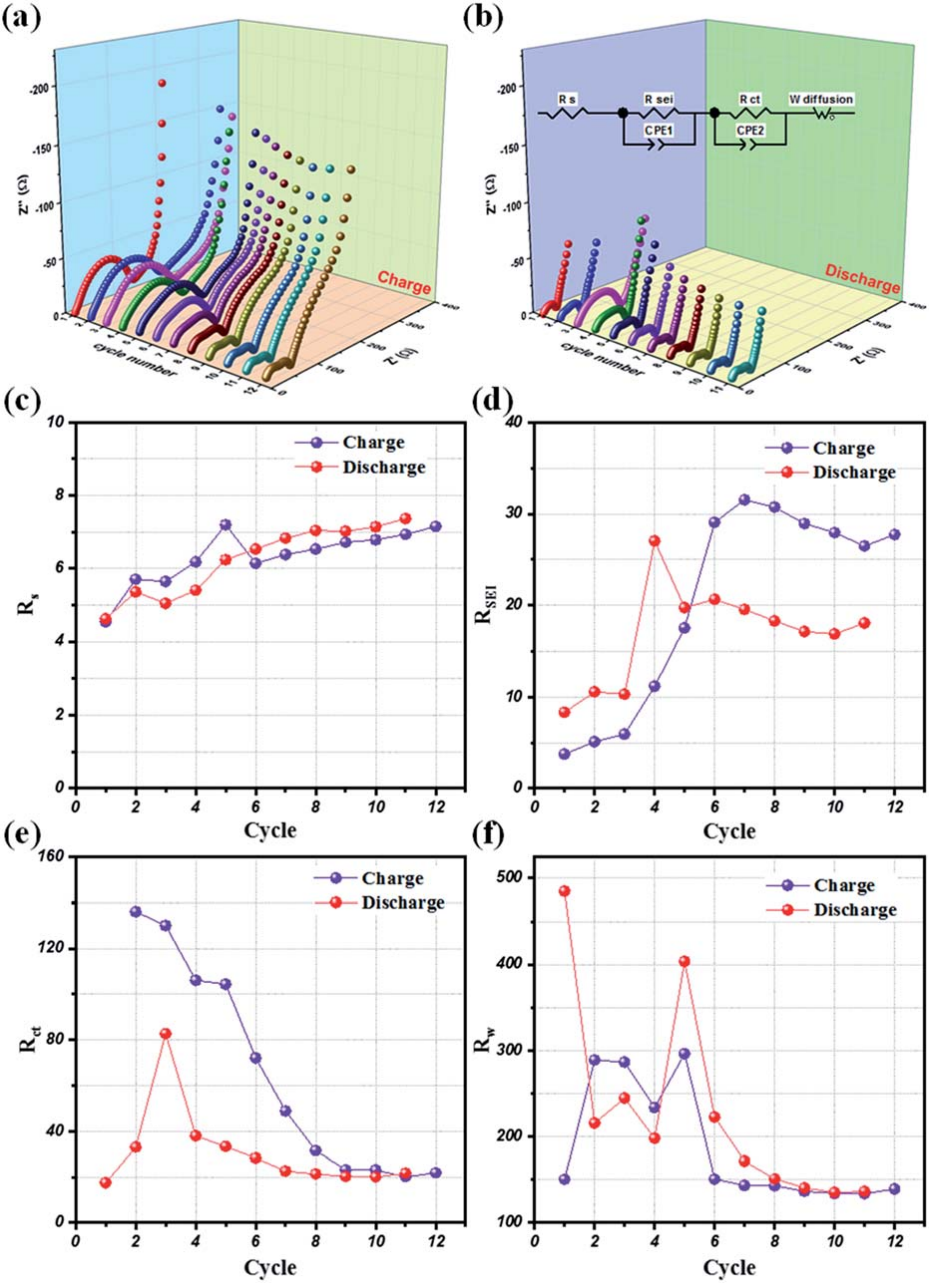
The variation of PEIS profiles at (a) 4.3 V (end of charge) and (b) 3.3 V (end of discharge) during cycling of 12 first cycles at 0.1C current density. Resistance variation of (c) *R*_S_, (d) *R*_SEI_, (e) *R*_CT_, and (f) Warburg elements in PEIS during cycling at 0.1C current density.

Potentio electrochemical impedance spectroscopy (PEIS) analysis was also conducted for an in-depth understanding of the reaction mechanism of LMO@C in a lithium test cell working within the designated potential range of 3.3 V to 4.3 V. Precisely, a series of *in situ* PEIS data were collected at the end of charge and discharge states, respectively, during the initial few galvanostatic cycles at 0.1C current density and the results are illustrated in Fig. 8(a) and (b). The compressed semicircle of charge state is dramatically reduced within 10 first cycles when the Li^+^ ions start to de-intercalate/intercalate from the structure, thereby helping to smoothen the reaction sites and increasing the electrical conductivity. From the subsequent cycle, the EIS spectra are slowly stabilized during the galvanostatic charge/discharge activation process. Meanwhile, the discharge EIS curves exhibit a stable behavior alongside the repeated cycling, demonstrating a sustainable and enduring spinel LMO crystal structure.

Further, an electrical equivalent circuit (EEC) was carefully studied using the Z-VIEW software, providing a closest analog description of the electrochemical kinetics. Accordingly, the EEC contained a resistor *R*_S_ in series with two parallel *R*_CT_ combinations (*R*_SEI_ + CPE_1_, *R*_CT_ + CPE_2_), and connected to a Warburg element, *W*, as shown in Fig. 8(b) (inset). According to the Barsoukov model and our previous reports,^52,58,59^ the EEC elements are described in the various stages of the lithium-ion insertion/exertion as follows: (i) *R*_S_ is attributed to the ion transport inside the separator and the electrolyte; (ii) the *R*_SEI_ + CPE_1_ combination represents lithium migration through the SEI layer; (iii) the *R*_CT_ + CPE_2_ group reflects the charge-transfer at the electrode/electrolyte interface; and (iv) *W* illustrates the Li^+^ ion diffusion inside the bulk phase of the active material. The variations of the *R*_S_, *R*_SEI_, *R*_CT_, and *W* values at the end of charge (4.3 V) and discharge conditions (3.3 V), respectively, for the initial 12 cycles are provided in Fig. 8(c)–(f). Interestingly, the SEI layer resistance (*R*_SEI_) slightly increases in the first few cycles, and then stabilizes during the subsequent cycles in both charge and discharge states (Fig. 8(d)). This reflects the formation of a protective SEI layer after a short activation, playing a key role in the highly stable electrochemical performance of the LMO electrode. Moreover, the charge transfer (*R*_CT_) and diffusion (*R*_W_) resistances drastically reduced for the initial few (∼8) cycles before showing negligent variation in the successive cycles. Therefore, these results clearly compliment the LMO/C cathode demonstrating a high rate performance, as observed from the galvanostatic results.

The enhanced electrochemical properties of the present material are presumably associated with the formation of the nanosized LMO/C particles and uniform carbon coating on the surface of the particles during the one-pot pyro-synthesis. More precisely, the nano-sized particles tend to increase the surface-area of the active particles and hence facilitate greater insertion and lithium storage capacity. The uniform carbon coating of the LMO nanoparticles tends to effectively reduce manganese dissolution in the electrolyte and also prevents volume expansion of the active material during repeated de-intercalation/intercalation reactions. Finally, the post-calcination procedure tend to contribute to the high crystallinity and stabilization of the LMO structure. Overall, the electrochemical performance of the present LMO/C cathode is competitive to those reported for LMO cathodes prepared by various combustion routes.^32,60–63^ Also, the energy storage capacity and retention capacity is comparable to those reported for a few doped-LMO cathodes of LIBs.^64,65^ This shows the effectiveness of the one-pot synthetic strategy in preparing highly crystalline nano-sized LMO/C cathodes with uniform carbon coating for appreciable performance in LIB applications. Although particle aggregation observed in the present synthesis need to be circumvented, more optimization of this method can be performed by varying the reaction conditions and the materials involved. In other words, we expect that the excellent results will further motivate the study of spinel LMO/C, with a focus on the effect of nanosizing and carbon coating on the surface of the particles. Moreover, this synthetic approach could be customized further to prepare a variety of oxide-based nanostructured electrodes for useful energy storage applications.

## Conclusion

Nanosized LMO/C composites were synthesized by a one-pot polyol-assisted pyro-synthesis followed by heat treatment at moderate temperatures. The XRD and electron microscopy results confirmed that the carbon coating and carbon network on the nanosized LMO particles was around 30 nm. The Rietveld refinement and XANES results revealed that the amount of Mn^3+^ ions is higher than that of Mn^4+^ ions in the LMO/C sample. Moreover, the synthesized LMO/C electrode exhibited excellent electrochemical properties: (1) the initial discharge capacity was 137.2 mA h g^−1^ with 86% and 81% initial capacity retention until 50 and 250 cycles at 0.1 and 1C, respectively, in the potential range 3.3–4.3 V, and (2) the initial discharge capacity was 289.95 mA h g^−1^ with 77.5% initial capacity retention until 50 cycles at 0.1C and 36% rate capability at 10C compared with that at 0.1C, in the potential range 2.5–4.3 V. The remarkable cycling ability and rate capability of the present cathode can be ascribed to the combined effect of the nano size of the particles and carbon coating. Detailed CV and EIS studies confirmed that the highly stable LMO structure formed by pyro-reaction clearly supports reversible Li-intercalation. In particular, the PEIS studies highlighted the active role of the formed SEI layer, after a short electrode activation, in facilitating a highly stable electrochemical reaction in the LMO/C electrode prepared by the present pyro-synthesis. Therefore, considering other reports on nanosized spinel LMO prepared by complex synthesis strategies, the present study offers a simple and cost-effective preparation technique to obtain nanosized carbon-coated spinel LMO with good electrochemical performance for LIB applications.

## Conflicts of interest

There are no conflicts to declare.

## Supplementary Material

RA-009-C9RA04015C-s001
