# Supplementary material for: One-pot pyro synthesis of a nanosized-LiMn2O4/C cathode with enhanced lithium storage properties
Source: RSC Adv. 2019 Aug 2;9(42):24030–8. doi: 10.1039/c9ra04015c (PMC9069505; doi:10.1039/c9ra04015c)
Supplement: RA-009-C9RA04015C-s001 [file RA-009-C9RA04015C-s001.pdf]

Electronic Supplementary Material (ESI) for RSC Advances.
This journal is © The Royal Society of Chemistry 2019

Supplementary Information

One-pot pyro synthesis of a nanosized-$LiMn_2O_4$/C cathode with enhanced lithium storage properties

Jeonggeun Jo[a], Sukyeung Nam[a], Seungmi Han[a], Vinod Mathew[a], Muhammad Hilmy Alfaruqi[a], Duong Tung Pham[a], Seokhun Kim[a], Sohyun Park[a], Sunhyun Park[a] and Jaekook Kim[a]*

[a]Department of Materials Science and Engineering, Chonnam National University, 300 Yongbong-dong, Bukgu, Gwangju 61186, Republic of Korea

---

*Corresponding author. Tel: +82-62-530-1703; Fax: +82-62-530-1699.

E–mail address: jaekook@chonnam.ac.kr (Jaekook Kim)

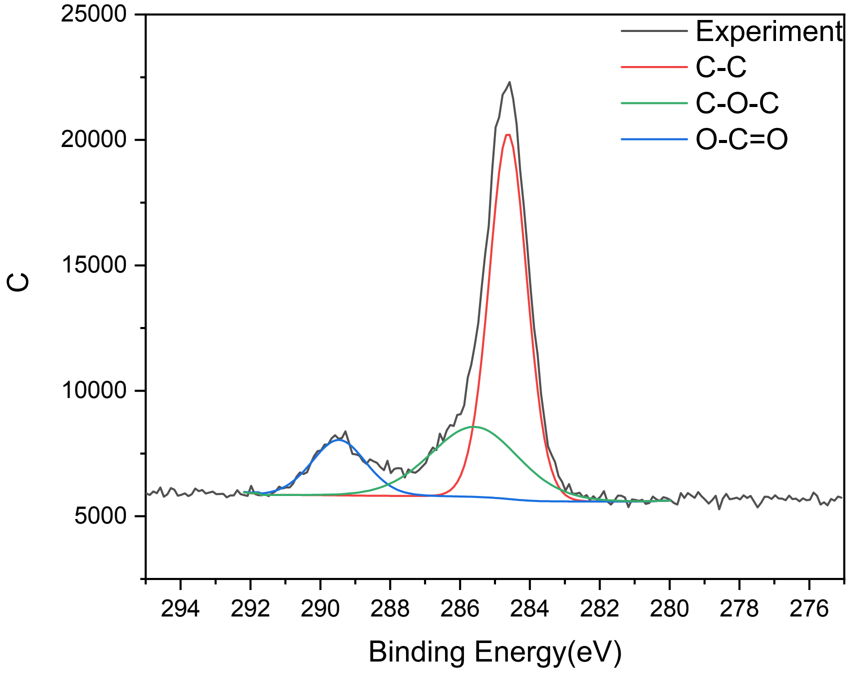

Fig. S1 C1s XPS spectra for the LMO/C sample prepared by the pyro-synthesis method.

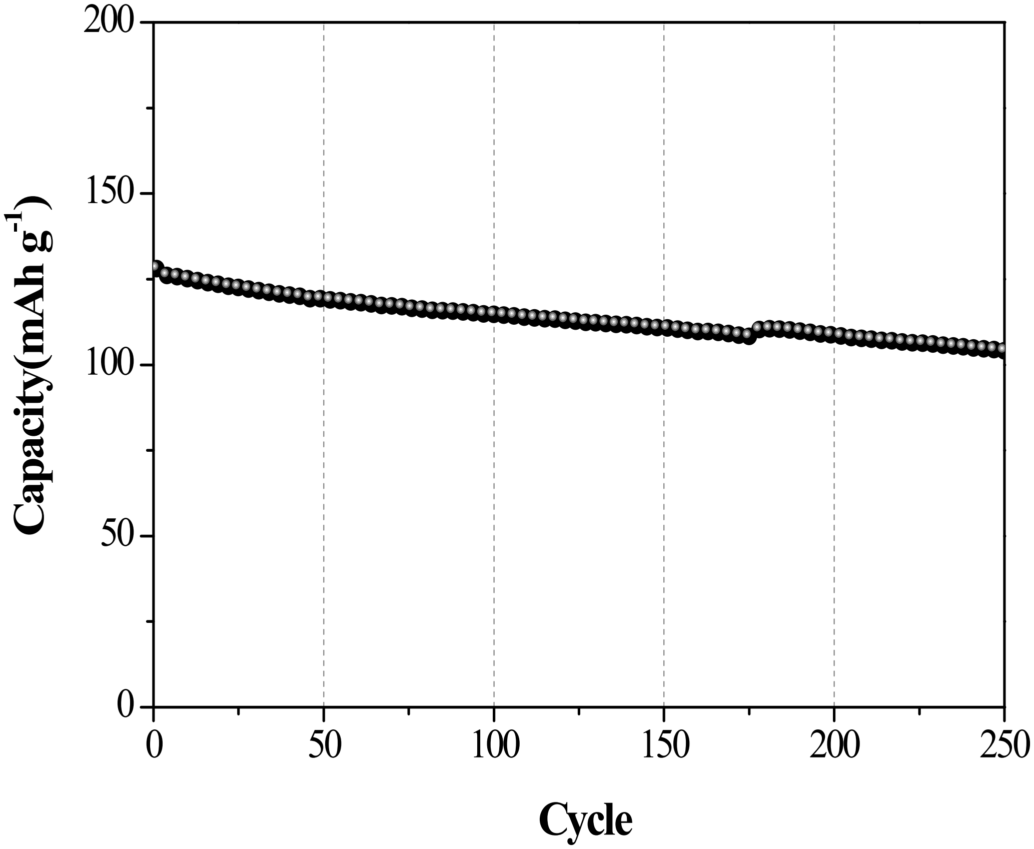

Fig. S2 Cycle performance of the nanosized LMO/C sample prepared by pyro synthesis at 1C current density in the voltage range 3.0 to 4.3 V.
